# Editorial: Empowering cancer care through immunonutrition

**DOI:** 10.3389/fnut.2026.1893379

**Published:** 2026-06-24

**Authors:** Laura J. Miller, Philip C. Calder

**Affiliations:** 1 Department of Nutrition and Dietetics, Nottingham University Hospitals NHS Trust, Nottingham, United Kingdom; 2 Centre for Rehabilitation and Ageing, School of Medicine, University of Nottingham, Nottingham, United Kingdom; 3 School of Human Development and Health, Faculty of Medicine, University of Southampton, Southampton, United Kingdom; 4 National Institute for Health and Care Research (NIHR) Southampton Biomedical Research Centre, University Hospital Southampton NHS Trust, Southampton, United Kingdom

**Keywords:** cancer, hematology malignant diseases, immunonutrition, immunotherapy, malnutrition, nutrition, postbiotic, prebiotic

Cancer incidence globally is expected to rise by an average of 63% by 2045 (1). Immunological factors such as chronic inflammation caused by cell senescence in aging (2, 3), obesity (4, 5), and infections (3, 6) are contributing to this rise. Despite this, the host immune system is being harnessed to develop next generation targeted cancer therapies, for example, check point inhibitors, CAR-T therapies, and vaccinations (7, 8). However, individual changes in host immune response (cellular competence and inflammation) and nutrient availability in the tumor microenvironment (TME) contribute to heterogeneity in treatment response (9, 10). Prolonged exposure to tumor antigens and immune stimulatory therapies, or overstimulation from chronic inflammation due to environmental factors (e.g., diet), can result in T-cell exhaustion (11). Checkpoint inhibitor drugs work partly by restoring exhausted T-cell function or blocking inhibitory receptors, for example, PD-1 or CTLA-4 (12, 13). However, cancer cells also consume nutrients and oxygen in the TME, adversely impacting T-cell metabolism and cellular recovery (14, 15).

An optimized nutritional state can support immunological resilience and recovery, but malnutrition is prevalent and impactful in people with cancer (16, 17). Malnourished patients exhibit enteric dysfunction with altered gut structure and function (18), and impaired cellular immunity, resulting in increased risk of infection (19). Current prophylactic antibiotic regimens during cancer treatment, while effective in reducing infections, increase potential for antimicrobial resistance, dysbiosis and side effects such as nausea and diarrhea.

Immunonutrition is the provision of nutrients, such as omega-3, certain amino acids and prebiotics, above basic nutritional needs to support immune-cell activity, or inflammatory pathways, to improve clinical outcomes. Studies of immunonutrient supplementation have shown promise in alleviating T-cell exhaustion (20) in cellular studies but some biological models show increased nutrient sensitivity in certain cancers (21). Supplementation trials in patients have shown variable effectiveness, with comparison between studies limited by heterogenous populations, treatments, doses, and outcomes (22, 23). So, further understanding of nutrient use by, and impact on, immune and cancer cells and of optimal dosing are essential for the safe and effective administration in patients.

This Research Topic “*Empowering cancer care through immunonutrition*” garnering five novel research studies, explores the interface between nutrition, immunology and cancer. Included papers provide examples from the laboratory to clinic, and from prognostics to interventions.

The first two studies explore the rapidly growing area of immune-nutrition biomarkers, prognostic tools to predict survival and inform stratified treatment or supportive interventions. Kim et al. explores the prognostic value of a known immune-nutrition biomarker, C-reactive protein (CRP)-albumin-lymphocyte (CALLY) index on overall survival in patients with surgically-treated non-small cell lung cancer (NSCLC). This composite biomarker integrates measures of inflammation, nutrition, and immunity, showing good survival prediction in other cancers (24, 25). This study showed close correlation of the index with overall survival but not relapse free survival.

Li et al., however, developed a novel prognostic biomarker by testing known blood-based measures of systemic inflammation, and nutritional status, for their greatest predictive value of survival, in adults with gastric signet ring cell carcinoma receiving curative surgery. The study found low lymphocyte-to-CRP ratio (LCR < 8,785.7) and geriatric nutritional risk index score (GNRI < 112.9) were independent predictors of poor overall survival, with increased discrimination when combined (LCR-GNRI; *p* < 0.001, C-index 0.76), particularly at 5-years.

The microbiome plays a role in immune modulation (26) via direct exposure to immune cells, or indirectly via microbiota-derived metabolites (post-biotics). Post-biotics, are non-viable microbial cells, their metabolites, and/or cell components, that modulate the immune system with health-promoting effects. Pre-biotic interventions such as oligosaccharides and pectins are selectively fermented by gut-microbes, synthesizing metabolites such as short-chained fatty acids (SCFAs, post-biotics). These SCFAs can integrate with G-coupled receptors, augmenting regulatory T-cells activity and aiding dendritic cell function (27, 28).

To harness the potential benefits of immune-modulating nutrition interventions studies exploring mechanisms of action and efficacy are required. In a study by Greier et al., organotypic slices cultures were taken from tumors removed from patients with head and neck squamous cell carcinoma (HNSCC), to explore the effect of SCFAs alone, or in combination with immunonutrients on the TME *in vitro*. The immunonutrient solution included glutamine, alanine, and fish oil as a source of omega-3s; the SCFA solution consisted of sodium-acetate, -propionate and -butyrate. The results showed heterogeneity in immune and tumor cell response to the immunonutrient composition and inter-patient strength of response. Cleaved caspase-3 (CC3) activity, a marker of apoptosis, was increased with SCFAs alone and decreased with the immunonutrient mix. However, inflammatory cytokine (TNFα and IL-1) suppression was greater with the immunonutrient mix. The caveat is that the research was done with tumor slices *ex vivo* which removes many steps of physiology seen *in vivo*.

The use of post-biotics and their immuno-regulatory effects are also being applied to symptom control during cancer treatment. Chen et al., conducted an RCT of JK5G post-biotics (inactivated lactic acid bacteria and their bioactive metabolites) exploring their effects on microbiota and metabolism and their potential to alleviate moderate to severe opioid-treated cancer-related pain. JK5G significantly decreased pain intensity scores compared to control, alongside increases in *Akkermansia muciniphila* and *Bifidobacterium* abundances, which were positively correlated with CD3+ and CD4+ T-cells, and negatively with pro-inflammatory cytokines such as IL-6 and TNF-alpha. Metabolomic analysis indicated that these beneficial effects may be associated with an upregulation of tryptophan and butanoate metabolism. While this was a mixed patient cohort, nearly 70% were lung cancer patients with a subgroup analysis showing greater strength of effect in this group.

Systematic implementation of nutritional screening, assessment and treatment is recommended for malnutrition prevention in people with cancer (29) but is often poorly applied (30). An RCT of structured vs. unstructured nutritional care pathways by Liu and Feng found structured screening, assessment and treatment (education and nutrition support) provided greater patient satisfaction, quality of life, and significant clinical improvements of nutritional outcomes. There was evidence of an anti-inflammatory trend in cytokine activity in the intervention group, but it was difficult to discern if the immunonutrition provision (subset of patients) contributed.

Together these papers highlight the key role nutrition plays in immune cell function and the potential for personalized immunonutrition to support next generation immune-modulating treatments in the fight for sustained response and improved symptom management in people with cancer.
